# Preoperative geriatric nutritional risk index and neutrophil-to-lymphocyte ratio relate to postoperative acute kidney injury in elderly patients undergoing laparoscopic abdominal surgery

**DOI:** 10.29219/fnr.v68.10564

**Published:** 2024-05-15

**Authors:** Hengchang Ren, Min Zhu, Hongli Yu, Yiqi Weng, Wenli Yu

**Affiliations:** Department of Anesthesiology, Tianjin First Central Hospital, Tianjin China

**Keywords:** acute kidney injury, preoperative Geriatric Nutritional Risk Index, Neutrophil-to-lymphocyte ratio, Laparoscopic abdominal surgery

## Abstract

**Background:**

Acute kidney injury (AKI) poses a significant concern in elderly patients undergoing laparoscopic abdominal surgery due to increased vulnerability arising from aging, comorbidities, and surgery-related factors. Early detection and intervention are crucial for mitigating short- and long-term consequences. This study aims to investigate the correlation between preoperative Geriatric Nutritional Risk Index (GNRI), neutrophil-to-lymphocyte ratio (NLR), and the occurrence of postoperative AKI in elderly patients undergoing laparoscopic abdominal surgery, as well as to assess the predictive value of their combined detection for postoperative AKI.

**Methods:**

A retrospective study involving 347 elderly patients (aged 60 years or older) undergoing laparoscopic abdominal surgery explored the relationship between preoperative GNRI, NLR, and postoperative AKI. GNRI was calculated based on serum albumin and body weight ratios, while NLR was derived from preoperative blood tests.

**Results:**

The combined GNRI and NLR test demonstrated superior predictive value (area under the curve [AUC] = 0.87) compared to individual markers. Multivariate logistic analysis identified age, American Society of Anesthesiologists (ASA) grade, comorbidities, preoperative GNRI, and NLR as independent risk factors for AKI. Correlation analysis affirmed a negative correlation between preoperative GNRI and AKI severity, and a positive correlation between preoperative NLR and AKI severity.

**Conclusion:**

The preoperative GNRI and NLR have clinical values in predicting postoperative AKI in elderly patients undergoing laparoscopic abdominal surgery.

## Popular scientific summary

- Elderly patients undergoing laparoscopic abdominal surgery can easily have acute kidney injury. Early detection and intervention is very important for such patients. The current study found that the preoperative Geriatric Nutritional Risk Index and neutrophil-to-lymphocyte ratio have clinical values in predicting postoperative acute kidney injury in these patients.

Acute kidney injury (AKI) is a clinical syndrome characterized by a rapid and often reversible decline in renal function, resulting in the accumulation of metabolic waste products and disruption of fluid and electrolyte balance (1, 2). AKI in elderly patients has been a significant clinical concern due to the higher prevalence of predisposing factors and the potential for adverse outcomes (3). The aging process itself, coupled with the presence of comorbidities, medications, and other age-related changes, increases the vulnerability of elderly individuals to AKI (4). Therefore, AKI can impact both short-term and long-term outcomes in elderly patients following laparoscopic abdominal surgery (5, 6). Early diagnosis and intervention are of paramount importance in preventing postoperative AKI in elderly patients undergoing abdominal surgeries, contributing to long-term implications for reducing societal and family healthcare expenditures (7).

The Geriatric Nutritional Risk Index (GNRI) is a valuable tool in assessing the nutritional status and predicting health outcomes in the elderly population (8). Beyond its predictive capabilities, the GNRI finds applications in various clinical scenarios, including preoperative risk assessment, chronic disease management, and monitoring response to nutritional interventions (9, 10). The Neutrophil-to-Lymphocyte Ratio (NLR) has emerged as a significant and easily accessible inflammatory marker with prognostic value in various medical conditions (11, 12). The NLR is a calculated ratio obtained by dividing the absolute neutrophil count by the absolute lymphocyte count, both routinely measured components of the complete blood count (13). Elevated NLR reflects an imbalance between pro-inflammatory neutrophils and anti-inflammatory lymphocytes, serving as a surrogate marker for systemic inflammation (14). Numerous studies across diverse medical specialties have demonstrated the prognostic value of NLR in predicting outcomes such as mortality, disease recurrence, and complications (15, 16).

The NLR has gained recognition as a biomarker for cancer prognosis and surgical treatment response (17). In elderly patients undergoing abdominal surgery, a significant correlation has been observed between preoperative GNRI reduction and adverse outcomes (18). The purpose of this study is to investigate the correlation between preoperative GNRI, NLR, and the occurrence of postoperative AKI in elderly patients undergoing laparoscopic abdominal surgery, as well as to assess the predictive value of their combined detection for postoperative AKI.

## Methods

### Participants

This study is a retrospective investigation that included a total of 347 eligible patients from June 2018 to June 2023. The clinical data of these patients were reviewed to assess the occurrence of AKI after surgery. Among the included patients, 98 experienced AKI, while 249 did not (No AKI, named NAKI). An informed consent was obtained from all patients enrolled in the study. This study was approved by Tianjin First Central Hospital.

### Inclusion criteria

The inclusion criteria are as follows: Elderly patients scheduled for laparoscopic abdominal surgery at our institution, aged 60 years or older, with American Society of Anesthesiologists (ASA) grades I–III.

### Exclusion criteria

The exclusion criteria are as follows: Patients with a history of chronic kidney disease, who had undergone preoperative renal replacement therapy, acute cardiovascular or cerebrovascular diseases, acute or chronic infections, significant organ failure, or hematologic disorders. Patients undergoing urological, ureteral, or renal interventions during surgery; those who developed AKI due to other reasons during the perioperative period; and those who died within 7 days postoperatively or had incomplete clinical data were also excluded.

### Diagnostic criteria for AKI

The diagnostic criteria for AKI are as follows: An increase in serum creatinine (Scr) by ≥ 26.5 μmol/L (0.3 mg/dL) within 48 h; Scr levels reaching or exceeding 1.5 times the baseline value within 7 days; urine output persistently below 0.5 mL/(kg·h) for 6 consecutive hours.

### Geriatric Nutritional Risk Index

The GNRI was devised by integrating two nutritional parameters: serum albumin levels and the ratio of actual weight to ideal body weight. Its formulation is expressed through the following equation: GNRI = 1.487 × serum albumin (g/L) + 41.7 × actual/usual body weight (kg). The calculation of ideal body weight is determined by the formula: ideal body weight (kg) = 22 × square of height (m^2^). In instances where the patient’s actual body weight exceeds the ideal body weight, the ratio of actual body weight to ideal body weight is considered as 1.

### Neutrophil-to-lymphocyte ratio

The preoperative measurements of neutrophils, lymphocytes, Scr, and other levels are obtained through preoperative fasting blood tests.

### Statistical analysis

Data analysis was conducted using software such as SPSS 26.0, MedCalc 15.0, and R 4.1.2. Normally distributed metric data are expressed as mean ± standard deviation (x ± s), and between-group comparisons were assessed using independent sample *t*-tests. Non-normally distributed metric data are presented as median (M) and interquartile range (IQR), with between-group comparisons performed using the Mann–Whitney *U* test. Count data were represented as frequencies (percentages), and between-group comparisons were evaluated using the chi-square test. Independent risk factors for AKI after abdominal surgery in elderly patients were analyzed using multiple-factor logistic regression. Receiver Operating Characteristic (ROC) curves were generated. A significance level of *P* < 0.05 indicates statistically significant differences.

## Results

### Analysis of single factors affecting the occurrence of AKI in elderly patients undergoing laparoscopic abdominal surgery

Table 1 presents an analysis of single factors influencing the occurrence of AKI in elderly patients undergoing laparoscopic abdominal surgery. The table provides information on various factors, including age, gender, type of surgery, ASA grade, comorbidities (coronary heart disease, diabetes mellitus, chronic obstructive pulmonary disease, hypertension, hyperlipidemia, hypoproteinemia, and hypohemia), as well as operation time, blood loss during operation, preoperative Scr, and preoperative estimated glomerular filtration rate (eGFR). Notable findings include a significant age difference between NAKI and AKI groups (*P* < 0.001), with AKI patients being older. ASA grade, a measure of overall health, demonstrated a significant association with AKI (*P* < 0.001). Specific comorbidities such as diabetes mellitus (*P* = 0.012), hypoproteinemia (*P* = 0.002), and hypohemia (*P* = 0.001) exhibited significant correlations with AKI. The analysis also extends to operative parameters, revealing differences in preoperative Scr levels (*P* = 0.007) and preoperative eGFR values (*P* = 0.015) between the two groups.

**Table 1:** Analysis of single factors affecting the occurrence of acute kidney injury in elderly patients undergoing laparoscopic abdominal surgery.

Factors	NAKI (*n* = 249)	AKI (*n* = 98)	*P*
Age (years)	68.83 ± 6.19	72.61 ± 7.43	< 0.001
Gender			
Male	147 (59.0%)	62 (63.3%)	0.543
Female	102 (41.0%)	36 (36.7%)	
Type of surgery			
Radical gastrectomy	73 (29.3%)	38 (38.8%)	0.404
Colorectal surgery	69 (27.7%)	24 (24.5%)	
Hepatobiliary	85 (34.1%)	29 (29.6%)	
Splenic	22 (8.8%)	7 (7.1%)	
ASA grade			
I	46 (18.5%)	5 (5.1%)	< 0.001
II	112 (44.9%)	37 (37.8%)	
III	91 (36.6%)	56 (57.1%)	
Coronary heart disease			
Yes	39 (15.7%)	22 (22.4%)	0.158
No	210 (84.3%)	76 (77.6%)	
Diabetes mellitus			
Yes	51 (20.5%)	33 (33.7%)	0.012
No	198 (79.5%)	65 (66.3%)	
Chronic obstructive pulmonary disease			
Yes	27 (10.8%)	16 (16.3%)	0.204
No	222 (89.2%)	82 (83.7%)	
Hypertension			
Yes	87 (34.9%)	41 (41.8%)	0.266
No	162 (65.1%)	57 (58.2%)	
Hyperlipidemia			
Yes	93 (37.3%)	44 (44.9%)	0.223
No	156 (62.7%)	54 (55.1%)	
Hypoproteinemia			
Yes	21 (8.4%)	21 (21.4%)	0.002
No	228 (91.6%)	77 (78.6%)	
Hypohemia			
Yes	84 (33.7%)	52 (53.1%)	0.001
No	165 (66.3%)	46 (46.9%)	
Operation time (min)	147.28 ± 33.09	155.92 ± 35.67	0.257
Blood loss during operation (mL)	243.34 ± 36.54	260.15 ± 39.29	0.184
Preoperative Scr (μmol/L)	73.14 ± 10.25	81.06 ± 11.17	0.007
Preoperative eGFR (mL/(min × 1.73 m^2^))	95.22 ± 16.63	87.49 ± 16.04	0.015

The data were shown as mean ± SD or n (percentage). The comparisons of data between the two groups were done by Mann-Whitney test, Unpaired *t* test with Welch’s correction, Fisher’s exact test, or Chi-square test.

### Comparisons of preoperative GNRI and NLR between the AKI and the NAKI group

**Fig. 1:**
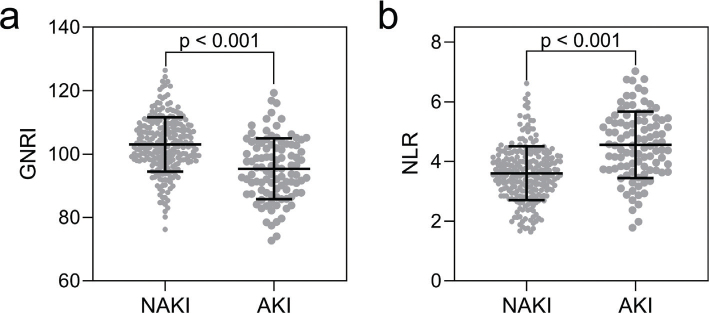
Comparisons of preoperative geriatric nutritional risk index (GNRI, a) and neutrophil-to-lymphocyte ratio (NLR, b) between acute kidney injury group (AKI, *n* = 98) and no acute kidney injury (NAKI, *n* = 249) in elderly patients undergoing laparoscopic abdominal surgery. The data were shown as mean ± SD. *P* values were calculated from Unpaired t test with Welch’s correction.

Figure 1 illustrates the comparisons of preoperative GNRI and NLR between the AKI group (*n* = 98) and the NAKI group (*n* = 249) in elderly patients undergoing laparoscopic abdominal surgery. The comparison of preoperative GNRI and NLR between the two groups revealed significant differences, with AKI patients exhibiting a significant decrease in preoperative GNRI (Fig. 1A, *P* < 0.001) and a notable increase in NLR (Fig. 1B, *P* < 0.001).

### Predictive values of preoperative GNRI, NLR, and their combined test for AKI in elderly patients undergoing laparoscopic abdominal surgery

**Fig. 2:**
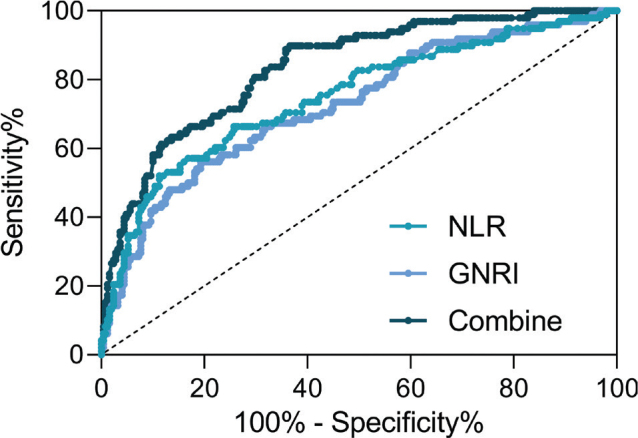
ROC analysis of predictive values of preoperative geriatric nutritional risk index (GNRI), neutrophil-to-lymphocyte ratio (NLR), and their combined test for acute kidney injury in elderly patients undergoing laparoscopic abdominal surgery.

Figure 2 depicts the ROC analysis evaluating the predictive values of preoperative GNRI and NLR, as well as their combined test, for AKI in elderly patients undergoing laparoscopic abdominal surgery. The ROC analysis revealed that the combined test, represented by COMPUTE Combine = – 0.1 * GNRI + 0.896 * NLR, exhibited the highest area under the curve (AUC) of 0.87 (95% CI: 0.83 to 0.91), surpassing the individual predictive values of preoperative GNRI (Fig. 2, AUC = 0.73, *P* < 0.001) and NLR (Fig. 2, AUC = 0.75, *P* < 0.001). Table 2 presents specific cut-off values, AUC, *P* values, sensitivity, specificity, and Youden index for each parameter. Notably, the combined test demonstrated improved sensitivity and specificity compared to individual tests, achieving an AUC of 0.87. It was further observed that there was a weak negative correlation between GNRI and NLR among all the elderly patients undergoing laparoscopic abdominal surgery (*n* = 347) (*r* = -0.14, *P* = 0.011, Supplementary Fig. S1).

**Table 2:** Predictive values of preoperative geriatric nutritional risk index (GNRI), neutrophil-to-lymphocyte ratio (NLR), and their combined test for acute kidney injury in elderly patients undergoing laparoscopic abdominal surgery.

	Cut off	AUC (95% CI)	*P*	Sensitivity (%)	Specificity (%)	Youden index
Preoperative GNRI	96.56	0.73 (0.67 to 0.79)	< 0.001	56.12	80.72	0.37
Preoperative NLR	4.13	0.75 (0.69 to 0.81)	< 0.001	66.33	74.30	0.41
Combine^*^	-	0.87 (0.83 to 0.91)	< 0.001	71.43	84.74	0.56

* COMPUTE Combine = – 0.1 * GNRI + 0.896 * NLR.

### Multivariate logistic analysis for the occurrence of AKI in elderly patients undergoing laparoscopic abdominal surgery

Furthermore, Table 3 presents the results of multivariate logistic analysis for factors influencing AKI occurrence, indicating the preoperative GNRI and NLR, along with other factors such as age, ASA grade, diabetes mellitus, hypoproteinemia, hypohemia, preoperative Scr, and preoperative eGFR. Using the occurrence of AKI as the dependent variable (NAKI = 0, AKI = 1) and considering the factors that showed significant differences in Table 1, along with preoperative GNRI and NLR, as independent variables, the table provides specific odds ratio (OR) values and *P* values. The results indicate that age, ASA grade, hypoproteinemia, hypohemia, preoperative Scr, preoperative GNRI, and preoperative NLR exhibit significant differences. This suggests that age, ASA grade, hypoproteinemia, hypohemia, preoperative Scr, GNRI, and NLR are independent risk factors for the occurrence of AKI in elderly patients undergoing laparoscopic abdominal surgery.

**Table 3:** Multivariate logistic analysis for the occurrence of acute kidney injury in elderly patients undergoing laparoscopic abdominal surgery.

	OR	95% CI	*P*
Age	1.242	1.083 to 1.427	0.029
ASA grade	1.836	1.337 to 3.094	0.005
Diabetes mellitus	1.301	0.942 to 2.173	0.146
Hypoproteinemia	2.256	1.438 to 4.253	0.002
Hypohemia	1.571	1.225 to 3.582	0.018
Preoperative Scr	1.295	1.046 to 2.117	0.041
Preoperative eGFR	1.184	0.973 to 1.691	0.095
Preoperative GNRI	0.836	0.791 to 0.924	0.009
Preoperative NLR	1.973	1.672 to 3.286	0.002

### Correlation analysis of Scr at diagnosis with preoperative GNRI and NLR

**Fig. 3:**
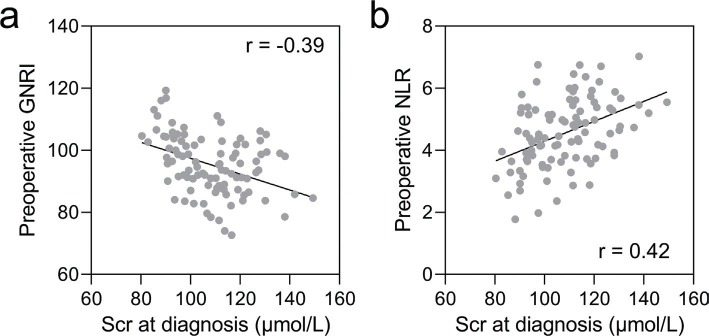
Pearson correlation analysis of serum creatinine at diagnosis with preoperative geriatric nutritional risk index (GNRI, a) and neutrophil-to-lymphocyte ratio (NLR, b). *P* < 0.001.

Figure 3 displays the results of Pearson correlation analysis examining the correlation between Scr levels at the time of AKI diagnosis and preoperative GNRI and NLR in the 98 patients diagnosed with AKI. The analysis demonstrates a negative correlation between preoperative GNRI and Scr levels (*r* = -0.39, Fig. 3A), and a positive correlation between preoperative NLR and Scr levels (*r* = -0.42, Fig. 3B), indicating that preoperative GNRI and NLR are correlated with the severity of AKI in elderly patients undergoing laparoscopic abdominal surgery.

## Discussion

The formidable challenge posed by an increasingly aging population has led to a steady rise in the proportion of elderly patients admitted to hospitals and undergoing surgical procedures. The physiological conditions of elderly patients, coupled with underlying medical conditions and abdominal surgeries, are major risk factors contributing to the elevated incidence and mortality rates of postoperative AKI (19). Elderly individuals, especially when affected by comorbid conditions, are more prone to developing AKI (20). Factors such as infections, heart failure, hypertension, gastrointestinal bleeding, surgical procedures, and urologic stones are predisposing elements that can easily induce AKI (21, 22). Therefore, early diagnosis and intervention hold paramount significance in preventing postoperative AKI in elderly patients undergoing abdominal surgeries, with far-reaching implications for reducing societal and family healthcare expenditures.

This study conducted a comprehensive investigation into the correlation between preoperative GNRI, NLR, and the occurrence of postoperative AKI in elderly patients undergoing laparoscopic abdominal surgery. The findings of this study shed light on the clinical significance of utilizing these biomarkers for risk assessment and the potential limitations inherent in their application.

This study delves into the comparisons of preoperative GNRI and NLR between the AKI and NAKI groups. The substantial decrease in preoperative GNRI and the notable increase in NLR among AKI patients are consistent with the study’s hypothesis and align with existing literature linking poor nutritional status and heightened inflammatory response to adverse postoperative outcomes, including AKI (23).

The ROC analysis, presented in Fig. 2, introduces a valuable dimension to the study by assessing the predictive values of preoperative GNRI and NLR individually and in combination. This finding is crucial for clinical applications, suggesting that a comprehensive assessment involving both nutritional status and inflammatory response can significantly improve AKI prediction. Comparisons with existing studies emphasizing the prognostic value of combined biomarkers reinforce the relevance of this approach in risk stratification (24).

Table 3 consolidates the multifactorial nature of AKI occurrence, employing multivariate logistic analysis. Age, ASA grade, comorbidities, and preoperative indicators, including GNRI and NLR, emerge as independent risk factors. The significance of age, ASA grade, and specific comorbidities aligns with the literature, highlighting the need for a holistic preoperative assessment. Importantly, the protective role of preoperative GNRI is noteworthy and adds a nuanced layer to risk stratification models. The findings parallel studies emphasizing the potential of nutritional interventions in improving surgical outcomes (25).

Figure 3 delves into the correlation between Scr levels at AKI diagnosis and preoperative GNRI and NLR. The observed significant correlation reaffirms the utility of these preoperative indicators not only in predicting AKI occurrence but also in gauging its severity. Comparable studies associating preoperative biomarkers with postoperative AKI severity corroborate the importance of early risk identification for targeted interventions.

The clinical significance of this study lies in its contribution to risk stratification and early identification of elderly patients at heightened risk of postoperative AKI. The aging population, coupled with comorbidities, makes this demographic particularly susceptible to AKI. The study’s focus on GNRI and NLR as predictive markers adds valuable insights into the preoperative assessment of nutritional status and inflammatory response, respectively. The observed correlation between reduced preoperative GNRI and increased NLR with postoperative AKI aligns with existing literature emphasizing the impact of nutritional status and inflammation on renal outcomes. The study’s multivariate logistic analysis further highlights the independent predictive value of these factors, affirming their role as crucial determinants of postoperative AKI in elderly patients. The incorporation of GNRI and NLR into routine preoperative assessments could potentially enhance risk stratification, allowing for timely interventions and improved patient outcomes (26).

However, certain limitations warrant consideration. First, the retrospective nature of the study may introduce selection bias and limits the establishment of causation. The reliance on historical clinical data may also lead to incomplete or inconsistent documentation, affecting the accuracy of the results. Additionally, the study’s single-center design may limit the generalizability of findings to a broader population. External factors, such as variations in surgical techniques or perioperative care practices, may influence AKI occurrence independently of GNRI and NLR. The exclusion criteria, although necessary to ensure data homogeneity, might exclude patients with specific characteristics that could influence AKI development. Moreover, while GNRI and NLR demonstrate promise as predictive markers, their utility may vary across different surgical procedures and patient populations.

## Conclusions

In conclusion, this study makes a valuable contribution to understanding the relationship between preoperative GNRI, NLR, and postoperative AKI in elderly patients undergoing laparoscopic abdominal surgery. The integration of these biomarkers into routine preoperative assessments could potentially enhance risk stratification and guide preventive strategies. Nevertheless, the study’s limitations highlight the need for further prospective, multicenter investigations to validate these findings and determine the broader applicability of GNRI and NLR in diverse clinical settings.

## Conflicts of interest and funding

The authors declare no conflict of interest. This study was supported by Tianjin Key Medical Discipline (Specialty) Construction Project (TJYXZDXK-045A).

## Authors’ contributions

The author’s contributions are as follows: HR, MZ, HY, YW, and WY: performed experiments and analyzed the data; HR, YW, and WY: contributed to the discussion of the data; WY: contributed to the supervision of the project, interpretation of the data, and writing the paper; and all authors read and approved the final manuscript.

## Supplementary Material


