# Supplementary material for: Preoperative geriatric nutritional risk index and neutrophil-to-lymphocyte ratio relate to postoperative acute kidney injury in elderly patients undergoing laparoscopic abdominal surgery
Source: Food Nutr Res. 2024 May 15;68:10.29219/fnr.v68.10564. doi: 10.29219/fnr.v68.10564 (PMC11167701; doi:10.29219/fnr.v68.10564)
Supplement: Supplementary file 1 [file FNR-68-10564-s1.docx]

Supplementary materials


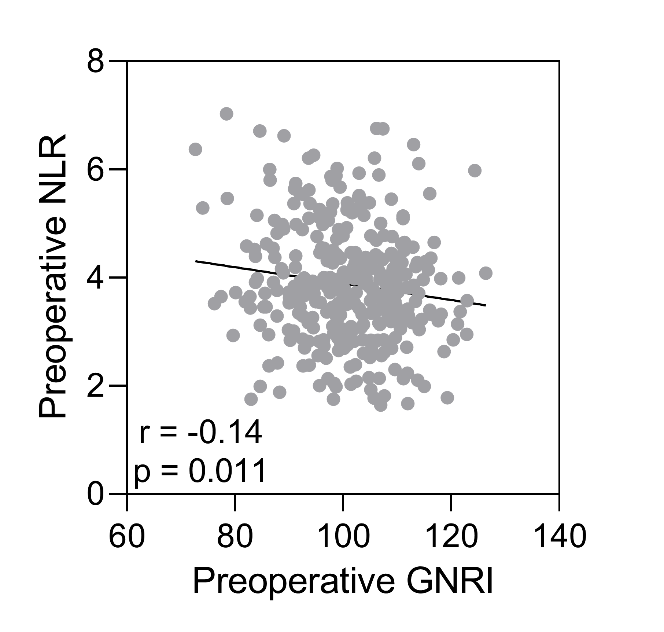


Figure S1. Pearson correlation analysis of preoperative geriatric nutritional risk index (GNRI) with neutrophil-to-lymphocyte ratio (NLR) in all elderly patients undergoing laparoscopic abdominal surgery (n = 347).
